# Yokukansan Alleviates Cancer Pain by Suppressing Matrix Metalloproteinase-9 in a Mouse Bone Metastasis Model

**DOI:** 10.1155/2019/2956920

**Published:** 2019-05-26

**Authors:** Kenta Nakao, Atsushi Fujiwara, Nobuyasu Komasawa, Denan Jin, Manabu Kitano, Sayuri Matsunami, Shinji Takai, Seiji Ito, Toshiaki Minami

**Affiliations:** ^1^Department of Anesthesiology, Osaka Medical College, 2-7 Daigaku-machi, Takatsuki, Osaka 569-8686, Japan; ^2^Department of Innovative Medicine, Graduate School of Medicine, Osaka Medical College, 2-7 Daigaku-machi, Takatsuki, Osaka 569-8686, Japan

## Abstract

Bone cancer pain control is difficult because it includes various characteristics of pain such as nociceptic and neuropathic pain. In this study, we investigated the effect of yokukansan (YKS), one of the traditional Japanese herbal medicines, on cancer pain in mouse bone metastasis model. Oral administration of YKS significantly alleviated pain behavior measured by quantitative body weight bearing. Furthermore, the pain behavior was also significantly alleviated by intrathecal and intraperitoneal administration of matrix metalloproteinase- (MMP-) 9 inhibitor, but not of MMP-2 inhibitor. MMP-9 expression was significantly elevated in the bone tissue on day 3 after carcinoma cell injection and in the ipsilateral spinal cord on day 7, which was suppressed by YKS administration. Taken together, these results suggest that YKS alleviates cancer pain via suppressing MMP-9 expression in bone metastasis model in mice.

## 1. Introduction

Common cancers such as those of the breast, lung, and prostate frequently metastasize to multiple sites in bones, where they can cause significant and intractable pain [1]. Similar to cancer itself, the factors that drive bone cancer pain evolve and change with disease progression [2]. Once cancer cells have metastasized to bone, they generate pain by releasing algogenic substances including protons, bradykinin, endothelins, prostaglandins, proteases, and tyrosine kinase activators [3]. The release of these factors by cancer cells can induce sensitization and activation of nerve fibers that innervate the bone. Additionally, these factors can drive a remarkable increase in the number, size, and activity of bone-destroying osteoclasts, which can ultimately result in fracture of the tumor-bearing bone [4]. Tumor growth in bone can also generate neuropathic pain by directly injuring nerve fibers as well as by inducing an active and highly pathological sprouting of both sensory and sympathetic nerve fibers that normally innervate the bone [5]. This structural reorganization of sensory and sympathetic nerve fibers in the bone, combined with the cellular and neurochemical reorganization that occurs in the spinal cord and brain, appears to contribute to the peripheral and central sensitization that is common in advanced bone cancer pain. These mechanistic insights have shown that bone cancer pain is a complex mixture of nociceptic and neuropathic types of pain produced by various cytokines and hormones [6].

Yokukansan (YKS) is a Japanese herbal medicine consisting of 7 main galenicals,* Atractylodes lancea*,* Poria sclerotium*,* Cnidium officinale*,* Angelica acutiloba*,* Bupleurum falcatum*,* Glycyrrhiza uralensis*, and* Uncaria rhynchophylla* [7]. It has been prescribed for the treatment of anxiety symptoms such as irritability, restlessness, and insomnia caused by delirium and by aggressive and dangerous behavior [7]. Furthermore, animal studies showed that YKS alleviates neuropathic pain [8] or morphine tolerance via the action of several pain-related molecules [9].

Matrix metalloproteinase- (MMP-) 9 and MMP-2 are reported to play a crucial role in neuropathic pain development [10]. MMP-9 is also related to various psychiatric disorders [11], which YKS alleviates effectively [12]. Based on these previous studies, we hypothesized that YKS may alleviate bone cancer pain.

To address this possibility, we evaluated the effect of YKS on cancer pain in a mouse model of bone metastasis. Here we demonstrated that YKS alleviated cancer pain by suppressing the expression of MMP-9.

## 2. Materials and Methods

### 2.1. Cell Culture

4T1 mouse mammary carcinoma cells were purchased from ATCC (ATCC® CRL-2539™, Manassas, VA, USA) and cultured in Dulbecco's modified Eagle medium (Gibco, Montreal, Quebec, Canada) supplemented with 10% heat-inactivated fetal bovine serum, 100 units/mL penicillin, and 100 *μ*g/mL streptomycin (Gibco). The cells were harvested by brief exposure to 0.25% (w/v) trypsin-ethylenediaminetetraacetic acid, washed 3 times with Dulbecco's modified Eagle medium, and suspended in the phosphate buffered saline (PBS) at 1×10^6^ cells/mL [13].

### 2.2. Animals

All experiments conformed to the regulations of the Animal Care Committee of Osaka Medical College (No. 26108) and received humane care in accordance with the guideline of the ethics committee of the International Association for the Study of Pain [14]. Four-week-old male Balb/C mice weighing 20-22 g were purchased from Japan SLC (Hamamatsu, Japan) and experiments of behaviors, immunohistochemistry, and RT-PCR were separately carried out in this study. The animals were housed under conditions of a 12 h light/12 h dark cycle, a constant temperature of 22 ± 2°C, and 60 ± 10% humidity. They were allowed free access to food and water before testing. Animal experiments were carried out so as to minimize the number of animals used and their suffering.

### 2.3. Bone Cancer Pain Model

A mouse model of bone cancer pain was established by intratibial injections of 4T1 cells. Briefly, after having been anesthetized with intraperitoneal (*i.p.*) injection of sodium pentobarbital (60 mg/kg), the right leg was shaved and the skin disinfected with 70% ethanol (v/v). A 0.5 cm long rostrocaudal incision was made in the skin over the upper one-third of the tibia with minimal damage to the underlying muscles and nerves. The right tibia was carefully exposed, and a 25-gauge needle was inserted into the medullary cavity of the bone through a hole drilled in the tibia. The needle was then removed and replaced with a long, thin blunt needle attached to a 10 *μ*L Hamilton syringe containing the 4T1 mouse mammary cancer cells suspended in PBS. Only suspensions consisting of single cells with >90% viability were used. The cells (1×10^4^ cells in 10 *μ*l PBS) were injected into the tibial bone cavity. After a 2 min delay to allow the cells to fill the bone cavity, the wound was finally closed with 1-0 silk threads [13].

### 2.4. Radiographic Evaluation of Bone Destruction

Radiographs of the right tibia were taken on the days 0, 3, 5, 7, and 10 after tumor injection by use of an X-ray machine, MX-70eco® (Medixtec Japan Corporation, Japan). Images were captured with mediXtec ImageView® (Medixtec Japan Corporation, Japan) to make a radiological assessment of the extent of the intraosseous lesions.

### 2.5. Behavioral Study

Pain-related behaviors were examined just before and 3, 5, and 7 days after the injection of the 4T1 breast cancer cells into the tibia and bone decompression. To quantify bone cancer pain objectively and quantitatively, we used a dynamic weight bearing (DWB) device (Bioseb, Boulogne, France), which consists of a plexiglass enclosure (W11×D11×H30 cm) with a floor sensor containing 1,936 pressure transducers.

The mouse was allowed to move freely for 5 min while a camera recorded each movement. The video recordings and pressure data relayed from the floor sensors were transmitted to a computer and stored for analysis. The transmitted pressure and visual data were synchronized by using the DWB software v1.3. All movements were recorded and validated by the experimenter in accordance with the position of the mouse on the device, indicating which paw corresponded to the set of pixels recognized by the sensors: right or left paw. A mean value for the weight (in grams) and area (in mm^2^) on every limb was calculated automatically for the whole testing period based on the length of time of each validated segment. Testicles and tail were excluded from the analysis. Animals were not acclimatized to the enclosure before the initial testing period in order to maximize exploration behaviors [15, 16].

### 2.6. Drug Administration

**Figure 1:**
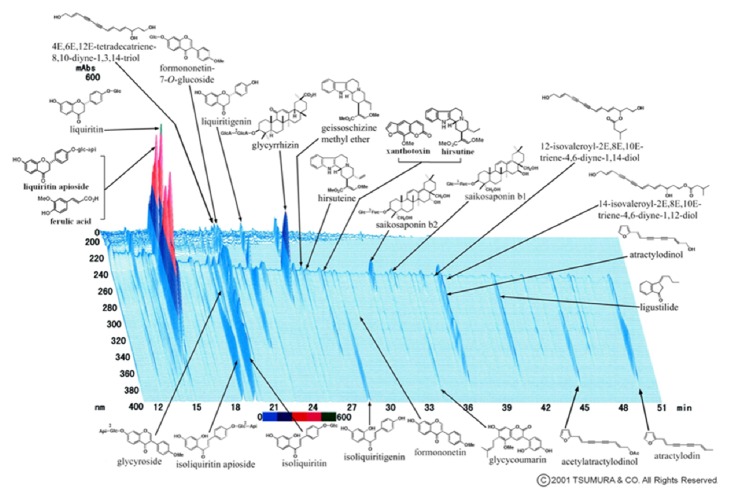
Ingredients of YKS used in this study. The figure shows a three-dimensional high-performance liquid chromatographic fingerprint of YKS used in this study. The chart was provided by Tsumura & Co.

YKS contained 7 dried medicinal herbs: 4.0 g rhizome of* Atractylodes lancea *De Candolle, 4.0 g sclerotium of* Poria cocos *Wolf, 3.0 g rhizome of* Cnidium officinale *Makino, 3.0 g root of* Angelica acutiloba *Kitagawa, 2.0 g root of* Bupleurum falcatum *Linné, 1.5 g root and stolon of* Glycyrrhiza uralensis *Fisher, and 3.0 g thorn of* Uncaria rhynchophylla *Miquel. The dry-powdered extracts of YKS (Lot Number: 2110054010) were supplied by Tsumura (Tokyo, Japan). The following active components had been identified by 3D-HPLC: 25 ingredients had been detected in YKS: ferulic acid; liquirtin apioside; liquirtin; 4E,6E,12E-tetradecatrien-8,10-diyne-1,3,14-triol; formononetin-7-O-glucoside; liquiritigenin; glycyrrhizin; geissoschizine methyl ether; hirsutein; xanthotoxin; hirsutin; saikosaponin b2, saikosaponin b1; 12-isovaleroyl-2E,8E,10E-triene-4,6-diyne-1,14-diol; 14-isovaleroyl-2E,8E,10E-triene-4,6-diyne-1,12-diol; atractylodinol; ligustilide; atractylodin; acetylatractylodinol; glycycoumarin; formononetin; isoliquiritigenin; isoliquiritin; isoliquiritin apioside; and glycycroside [17] (Figure 1). YKS (10 mg/0.1 mL) was daily administered orally to control and cancer-bearing mice for 7 days. The dosage of YKS used in this study was chosen on the basis of a previous report [8] and our preliminary studies.

We purchased MMP-9 inhibitor I (CAS 1177749-58-4) and MMP-2 inhibitor I (CAS 10335-69-0) from Calbiochem (La Jolla, CA, USA). Mice were injected* i.p*. and intrathecally (*i.t.*) with 50 *μ*g and 0.5 *μ*g of the inhibitor of MMP-9 or MMP-2, respectively, on day 7 after tumor cell injection according to the previous report [10] and our preliminary studies.

### 2.7. Real-Time Polymerase Chain Reaction (RT-PCR)

Three mice were randomly selected at each phase for dissecting the spinal cord. Total RNA (1 *μ*g) prepared from the spinal cord was transcribed into cDNA with Superscript III reverse transcriptase and random hexamers (Invitrogen, Carlsbad, CA, USA). Levels of mRNA were measured by RT-PCR using TaqMan fluorogenic probes on a LightCycler with software (Roche Diagnostics, Tokyo, Japan). All primers and probes for RT-PCR of MMP-2, MMP-9, and glyceraldehyde-3-phosphate dehydrogenase (GAPDH) were designed by Roche Diagnostics. The primers were 5′-gtgggacaagaaccagatcac-3′ (forward) and 5′-gcatcatccacggtttcag -3′ (reverse) for MMP-2, 5′-gaatgccccattctgcac -3′ (forward) and 5′-gggtttagtttctgcaaactgc -3′ (reverse) for MMP-9, and 5′-tgtccgtcgtggatctgac -3′ (forward) and 5′-cctgcttcaccaccttcttg-3′ (reverse) for GAPDH. The probes were 5′-gacctgga-3′ for MMP-2, 5′-ctccttcc-3′ for MMP-9, and 5′-cctggaga-3′ for GAPDH. mRNA levels of MMP-2 and MMP-9 were normalized to that level of GAPDH [18].

### 2.8. Histological Analysis

The mice used for histological analysis were different from those used in behavioral study. Six mice were selected randomly at each condition for extracting spinal cord. Tissue specimens were dissected from the spinal cord and fixed with Carnoy's fixative in 10% methanol overnight. The fixed tissues were embedded in paraffin and then cut at a thickness of 5 *μ*m. The sections were mounted on silanized slides (Dako, Carpinteria, CA, USA) and deparaffinized with xylene and ethanol. To retrieve the antigen prior to immunohistochemical staining, the sections were pretreated in 10 mmol/L citrate buffer and autoclaved for 5 min at 121°C.

For the detection of MMP-2 and MMP-9, the sections were incubated for 1 h at room temperature with anti-human MMP-2 and anti-human MMP-9 antibodies (Santa Cruz Biotechnol., Dallas, TX, USA). Then, they were reacted with 3-amino-9-ethylcarbazole reagent supplied in the labeled streptavidin-biotin peroxidase kit (LSAB kit, Dako). The sections were faintly counterstained with hematoxylin [19].

### 2.9. Statistics

Statistical analysis of the data was performed by using the unpaired two-sided Student's t-test or one-way repeated analysis of variance (ANOVA). Statistical analysis was performed utilizing JMP® 11 (SAS Institute Inc., Cary, NC, USA). Results were expressed as means ± standard deviation (SD), and differences were considered statistically significant at a P value of less than 0.05.

## 3. Results

### 3.1. Bone Cancer Pain in Breast Cancer Metastasis Model

**Figure 2:**
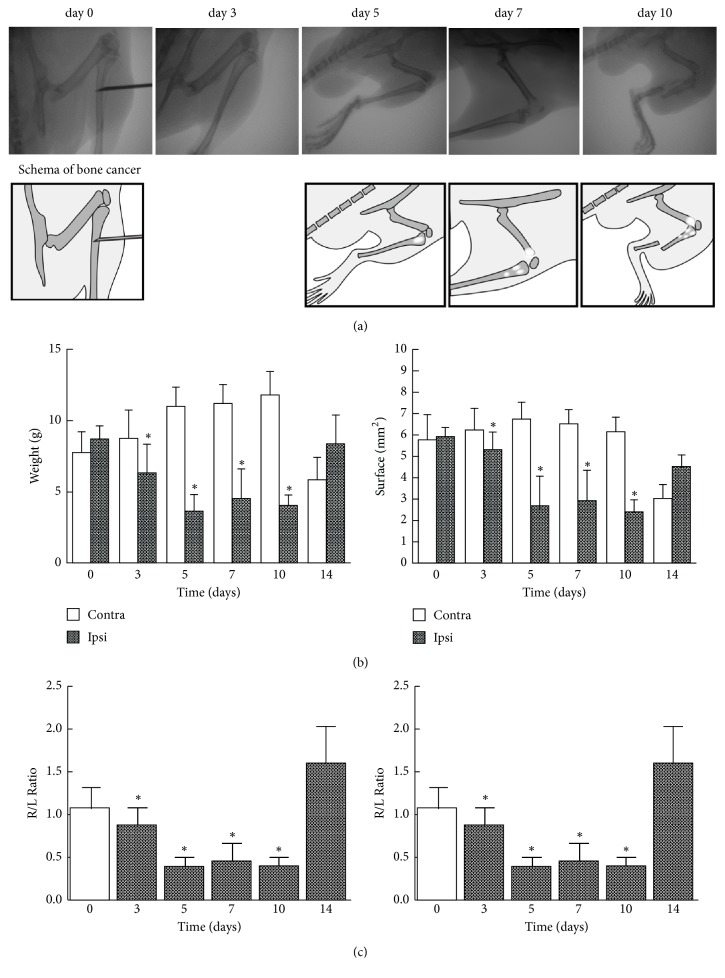
Preparation of bone cancer pain mice and time courses of cancer pain as assessed by weight bearing and surface resting of hind paws. (a) Radiographs of progressive bone destruction. These images are representative of the stages of bone destruction in the right tibia. (b, c) Animal body weight bearing and surface resting of right and left hind paws were measured for 5 min on the indicated days with the DWB device as described in “Materials and Methods.” Day 0 represents the presurgical baseline data. The data are expressed as the mean ± SD (n = 6 except for n =4 on day 14). One-way repeated ANOVA was applied for statistical analysis. *∗*P < 0.05 compared to left paw (b); *∗*P < 0.05 compared to the R/L ratio of day 0 (c) [label ordinate for the right-hand graph].

As reported previously [13], intrafemoral injection of 4T1 breast cancer tumor cells resulted in tumor growth with accompanying bone osteolysis and local invasion into the surrounding soft tissue day by day (Figure 2(a)). Radiographs of the right tibia showed the progressive loss of bone caused by tumor growth. On day 3, there was no apparent change; on day 5, there was minor loss of bone; on day 7, there were multiple osteolytic lesions; and on day10, there was bone fracture. Because 2 mice died on day 12, the results for day 14 were for the 4 surviving mice.

Using the DWB device, we analyzed body weight bearing and surface resting on the floor of hind paws. Before cancer cell injection, the mice distributed weight more to their hind paws than to their forepaws, and the weight of each hind paw was 8-9 g without laterality. Weight bearing of the tumor-injected right paw was significantly reduced compared with that of the left one on days 3, 5, 7, and 10 (P<0.05; Figure 2(b) and Sup. Table 1). When the weight bearing was expressed as the ratio of right to left paw (R/L ratio), it became evident that the bone cancer pain started on day 3, reached its maximum on day 5, and lasted on day 10. The R/L ratio for the surface attachment of the paw on the floor also showed the same time course as that of weight bearing (P < 0.05; Figure 2(b) and Sup. Table 1). These results demonstrated that bone cancer pain decreased the use of their affected limb for exploring and weight bearing. On the other hand, this difference was diminished on day 14, possibly because bone and nerve structures had been destroyed by the overgrowth of the tumor. So we chose day 7 for subsequent evaluation of the effects of drugs on weight bearing and surface resting in the bone cancer model.

### 3.2. Effect of Oral YKS on Bone Cancer Pain

**Figure 3:**
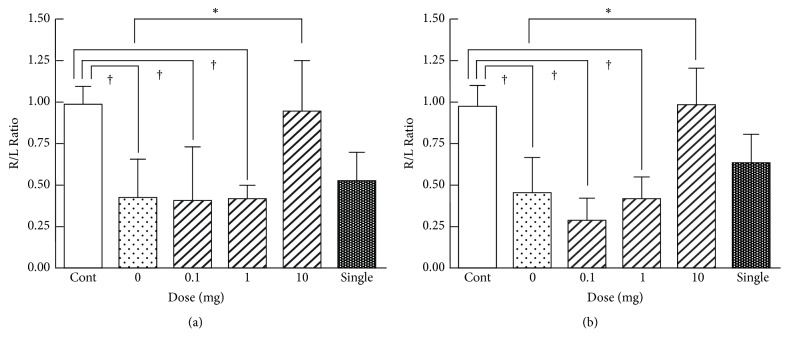
Dose dependencies of YKS for the effect on bone cancer pain. Mice were administered p.o. with the indicated doses of YKS once daily for 7 days or with a single p.o. administration of 10 mg YKS* 1 h* before assessment on day 7. Each column represents the R/L ratio of weight bearing (a) and surface resting (b). The values shown are the mean ± SD (n = 6). One-way repeated ANOVA was applied for statistical analysis. *∗*P < 0.05 compared to 0 mg (saline). †P < 0.05 compared to control.

To examine whether bone cancer pain could be alleviated by YKS, on day 7 we examined the effect of various doses of YKS administered orally and daily. Daily administration of YKS significantly increased the R/L ratio of weight bearing (Figure 3(a) and Sup. Table 2) and surface resting (Figure 3(b) and Sup. Table 2) in the animals when given at 10 mg, but not at 0.1 or 1 mg. A single administration of YKS at 10 mg appeared to increase the ratio, but its effect was not significant (Figures 3(a) and 3(b) and Sup. Table 2).

**Figure 4:**
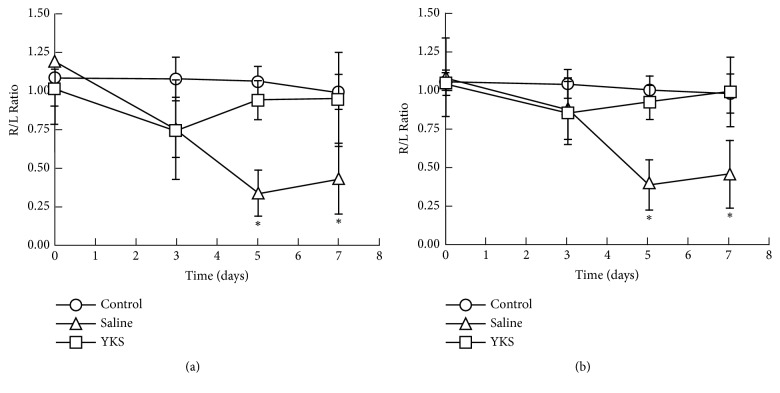
Effect of daily oral YKS on bone cancer pain. Mice injected by 4T1 cells and uninjected control mice were p.o. administered with 10 mg of YKS or saline once daily for 7 days. Weight bearing (a) and surface resting (b) on right and left hind paws were measured on the indicated days with the DWB device as described in the legend for Figure 2. The values shown are the mean ± SD (n = 6). Student's t-test was applied for statistical analysis. *∗*P < 0.05 compared to saline administration.

Next we examined the effect of 10 mg YKS on the progression of cancer pain. Daily administration of YKS significantly returned the R/L ratio of weight bearing (Figure 4(a) and Sup. Table 3) and surface resting (Figure 4(b) and Sup. Table 3) to 1.0 on day 5 and day 7 (P < 0.05). However, YKS did not affect the behavior of normal mice (Figures 4(a) and 3(b) and Sup. Table 3).

### 3.3. Effect of MMP-2 or MMP-9 Inhibitor on Bone Cancer Pain

MMP-2 and MMP-9 have been reported to play distinct roles in the development of neuropathic pain [20]. Local inhibition of MMP-9 via the intrathecal route inhibits the early phase of neuropathic pain, whereas that of MMP-2 suppresses its late phase [10].

**Figure 5:**
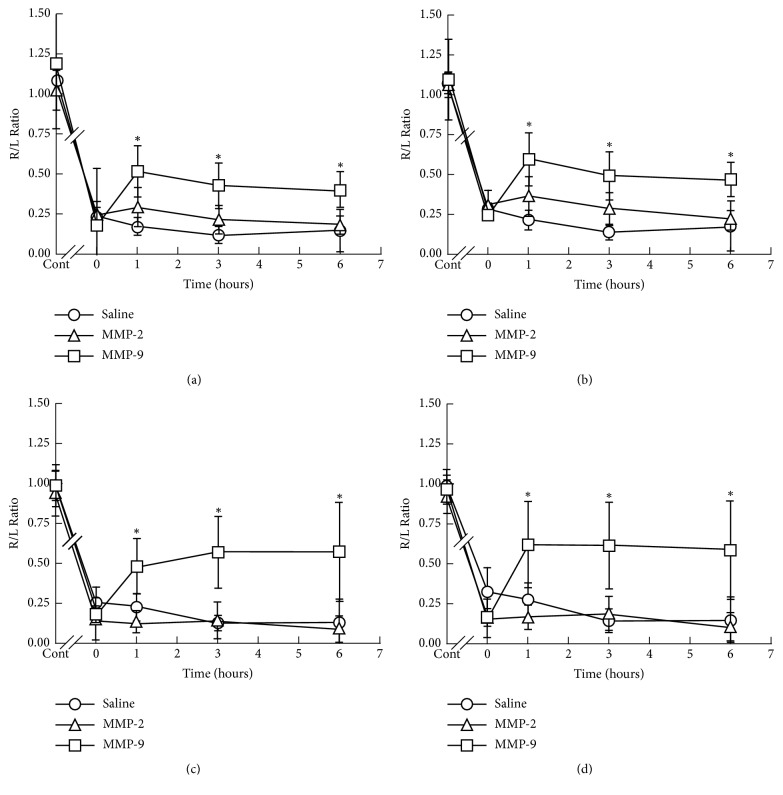
Time courses for the effect of* i.t.* or* i.p*. injection of saline, MMP-2 inhibitor or MMP-9 inhibitor on bone cancer pain. Bone cancer model mice were* i.p* injected with 50 *μ*g of MMP-2 or MMP-9 inhibitor (a, b) or* i.t*. injected with 0.5 *μ*g of MMP-2 or MMP-9 inhibitor (c, d); and weight bearing (a, c) and surface resting (b, d) were measured as described in the legend for  Figure 2. The values shown are the mean ± SD (n=6). Student's t-test was applied for statistical analysis. *∗*P < 0.05 compared to saline.

Therefore, next we examined the effect of inhibitors of MMP-2 and MMP-9 injected* i.p*. or* i.t.* on weight bearing and surface resting on day 7 in the model mice. The reduced R/L ratio of weight bearing (Figure 5(a) and Sup. Table 4) and surface resting (Figure 5(b) and Sup. Table 4) was partially and significantly reversed at 1 h after either the* i.p.* or* i.t.* injection of MMP-9 inhibitor, and this reversal continued for 6 h. In contrast, MMP-2 did not improve the R/L ratio of the mice by either* i.t.* or* i.p.* administration throughout the time course.

### 3.4. MMP-2 and MMP-9 Expression Levels in Bone Cancer Pain Model

**Figure 6:**
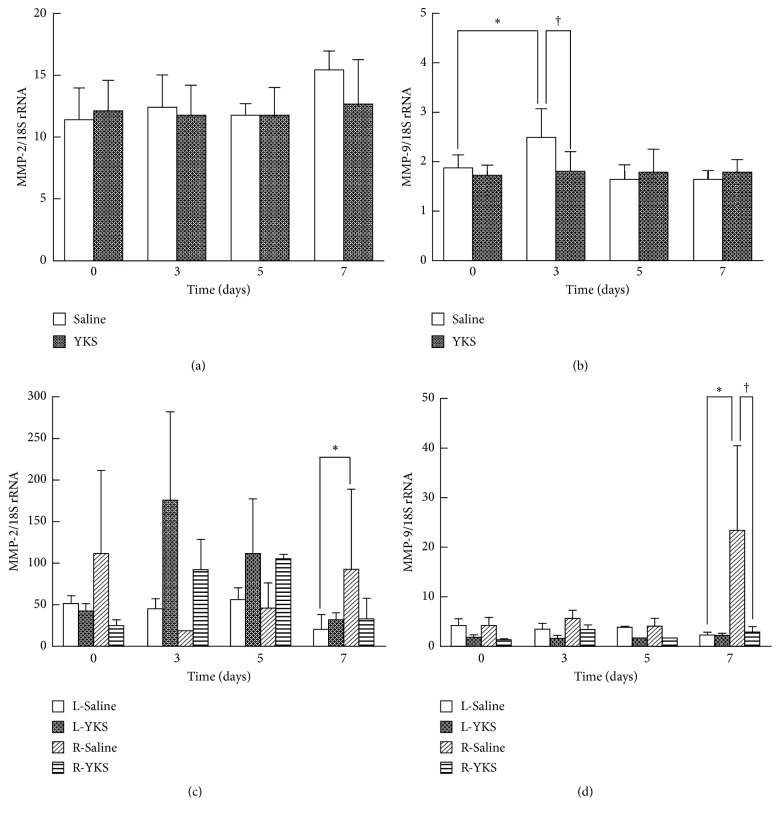
RT-PCR analysis of MMP-2 and MMP-9 expression levels. YKS (10 mg) or saline was p.o. administered once daily to mice injected with 4T1 cells, and the right tibia (a, b) and the left and right halves of spinal cord (c, d) of the mice were dissected on the indicated days. RT-PCR was carried out as described in “Materials and Methods.” mRNA expression of MMP-2 (a, c) and MMP-9 (b, d) was normalized to that of GAPDH, and the data are expressed as the mean ± SD (n=3). Student's t-test was applied for statistical analysis. *∗*P < 0.05 compared to day 0 (b) or to left halves of spinal cord (d). †P < 0.05 compared to YKS.

RT-PCR showed a significant increase in MMP-9 expression in the tumor-injected right tibia on day 3, whereas no significant difference or tendency was seen in the MMP-2 expression. YKS administration significantly suppressed the MMP-9 expression at the right tibia at the point of day 3 (P < 0.05). In contrast, MMP-2 expression was not significantly affected by the YKS administration throughout the time course (Figures 6(a) and 6(b) and Sup. Table 5).

In addition, RT-PCR showed a significant increase in MMP-9 expression in the right spinal cord during bone tumor progression, especially on day 7, whereas no significant difference or tendency was found for the MMP-2 expression. YKS administration significantly suppressed the MMP-9 expression at the right-side spinal cord at the point of day 7 (P < 0.05). In contrast, MMP-2 expression was not significantly affected by the YKS administration during the time course (Figures 6(c) and 6(d) and Sup. Table 5).

**Figure 7:**
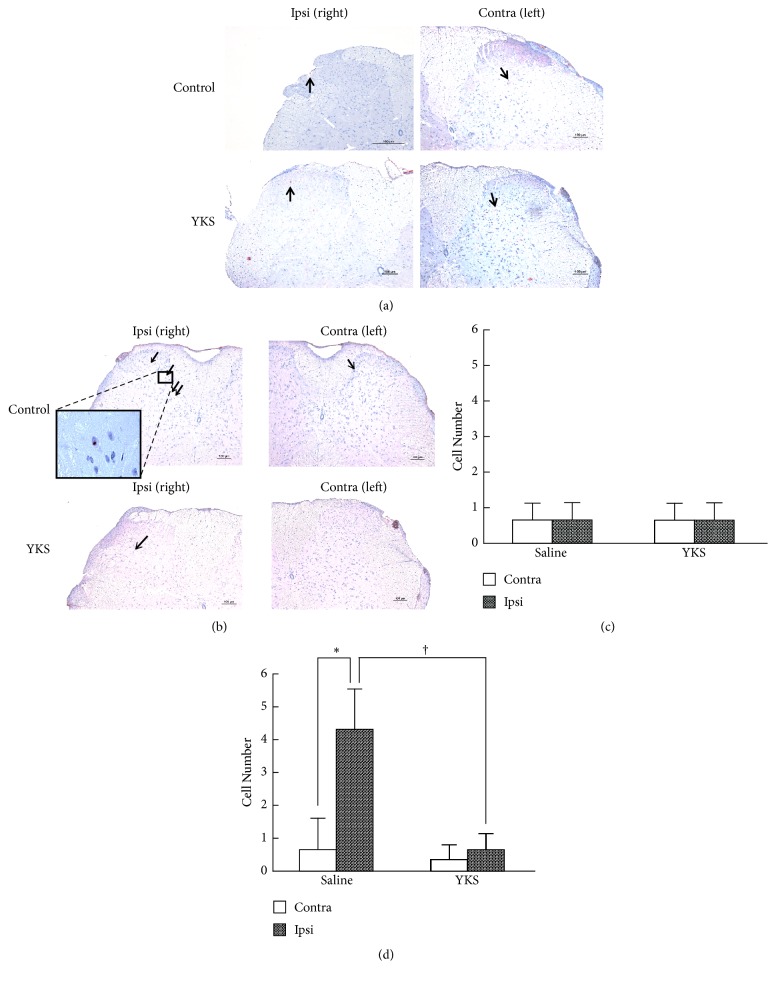
MMP-9 and MMP-2 immunostaining in the spinal cord. (a, b) Immunostaining of MMP-2 (a) and MMP-9 (b) in the dorsal horn of the spinal cord. The lumbar spinal cord (L4-L5) was prepared from cancer pain model mice on day 7 following p.o. administration of saline or YKS (10 mg) once daily. Transverse sections (40-*μ*m thick) were immunostained with anti-MMP-2 and MMP-9 antibodies as described in “Materials and methods.” The sections were counterstained with hematoxylin. Scale bars, 100 *μ*m (a, b). Arrows indicate immunopositive cells. (c, d) The number of MMP-2- (c) and MMP-9- (d) positive cells in the dorsal horn. The number of immunopositive cells was counted in 4 sections for each treatment. The data are expressed as the mean ± SD (n = 4). Student's t-test was applied for statistical analysis. *∗*P<0.05, compared to left part. †P < 0.05 compared to YKS.

Figure 7 shows the results obtained for immunostaining of MMP-2 (a) and MMP-9 (b) in the spinal cord in the YKS- or saline-treated bone cancer model mice at day 7. The number of MMP-9-positive cells was significantly higher in the right dorsal horn than in the left one of the saline-treated mice, and this number was significantly diminished in the YKS-treated group (Figure 7(d) and Sup. Table 6). In contrast, the number of MMP-2-positive cells (Figure 7(a) and Sup. Table 6) did not differ between the right and left dorsal horn. Also, the number of these cells in the right dorsal horn was not changed significantly by YKS administration (Figure 7(c) and Sup. Table 6).

## 4. Discussion

Cancer-induced bone pain is a mixed-mechanism pain state exhibiting elements of both neuropathic and inflammatory pain, but with distinctive modifications made to the tissue and nerves in the periphery as well as unique neurochemical changes at the spinal cord level [6]. As it is often difficult to evaluate the cancer pain animal model due to its complex nature, we chose to adopt a weight- bearing measurement, by which we could objectively assess the weight and surface area of tumor and nontumor feet of the mice. On day 3-10 after the operation the mice developed abnormal gait, posture, and guarding behavior of the hind paw on the right-side ipsilateral to the 4T1 breast cancer cell injection into the tibia when placed on the DWB device. The time course of body weight bearing was quite similar to that for the surface resting in this bone cancer pain model (Figure 2 and Sup. Table 1). Our study demonstrated that this DWB device was an effective measurement system to evaluate bone cancer pain in mice.

MMPs, also referred to as matrixins, hydrolyze components of the extracellular matrix. These proteinases play a central role in biological processes, such as embryogenesis, normal tissue remodeling, angiogenesis, cancer progression, and pain signal transduction. MMP-9 plays an important role in various aspects of homeostasis and pathogenesis of cancer development and in the mechanism of pain [10]. Ji et al. (2009) demonstrated distinct roles of MMP-9 and MMP-2 in neuropathic pain development: (i) transient MMP-9 upregulation after nerve injury is critical for the early-phase development of neuropathic pain; (ii) sustained MMP-2 upregulation maintains neuropathic pain; and (iii) MMP-9 and MMP-2 induce the cleavage of interleukin-1*β* for its activation in the early and late phase of nerve injury, respectively [20]. Furthermore, acute morphine treatment induces rapid and transient MMP-9 upregulation in primary sensory neurons to counteract opioid analgesia [21]. The intraoperative infusion of remifentanil, a potent, short-acting synthetic opioid analgesic drug, elicits MMP-9 activation, and upregulation in primary sensory neurons and subsequently causes interleukin-1*β* cleavage in dosal root ganglia, leading to central sensitization in the spinal cord to induce postoperative hyperalgesia [22]. Also, the transient increase in MMP-9 protein expression and subsequent continuous augmentation of MMP-9 activity in the midbrain (but not in the spinal cord) contributes to the development of morphine tolerance after repeated administration of morphine [23]. MMP-9 upregulation in the spinal cord has also been implicated in chronic opioid-induced dependence, tolerance, and withdrawal syndrome through possible neuronal activation and interaction with NMDA receptors (NR1 and NR2B) via integrin-*β*1 and nitric oxide pathways [24].

In our study, MMP-9 was associated with cancer pain, but MMP-2 was not. In our cancer pain model, the mice had a short life, and so they may have died before MMP-2 could increase, and bone destruction progresses more in approximately ten days, which is associated with not only neuropathic pain but also nociceptive pain. Unfortunately, YKS may not suppress the progress of bone cancer because that the mice administrated YKS daily also had a short life and the tumor growth assessed radiographically progressed day by day in the YKS-treated group and in the saline-injected group in a similar manner.

Several reports have shown that YKS exerts versatile actions via serotonergic, glutamatergic, cholinergic, dopaminergic, adrenergic, and GABAergic neural systems and that it exhibits neuroprotective, anti-stress, and anti-inflammatory effects and promotes neuroplasticity [17]. There are reportedly 2 main actions of YKS. First, YKS exerts an inhibitory effect on the glutamic acid transduction system [25, 26]. Secondly, YKS acts as a partial agonist of the serotonin_1A_ receptor and downregulates the serotonin_2A_ receptor [27]. YKS partially agonizes this receptor, even with a single dose and downregulates it with repeated doses. These actions are considered to account for the suppression of aggressive symptoms of delirium or dangerous behaviors.

Our results first indicated that MMP-9 played an essential role in the cancer bone pain in the model mice (Figure 5 and Sup. Table 3) and that its expression was significantly elevated in the ipsilateral bone tissue in the early phase and in dorsal horn of the spinal cord in the late phase of pain development, in which elevation was suppressed by YKS administration (Figures 6 and 7 and Sup.Tables 5 and 6). Furthermore, Nakagawa et al. (2012) previously reported that YKS and its active constituents inhibit morphine tolerance and physical dependence and that the latter is in part due to the prevention of the decreased membrane expression of the *α*_2A_-adrenoceptor in the brainstem by its prolonged blockade [9]. Bone cancer pain has been reported to have unique mechanisms and is resistant to morphine treatment. At present, a number of approaches are being aimed at reducing the level of bone cancer pain. YKS, acting via the inhibition of MMP-9, might provide a new candidate for the prevention and treatment of cancer pain as well as reduce opioid-induced unwanted side effects (such as opioid-induced hyperalgesia, dependence, tolerance, and withdrawal syndrome).

YKS is a versatile herbal remedy with a variety of neuropharmacological effects, and it may operate as a multicomponent drug having various active ingredients. In future studies, it will be important to assess the effective site of the palliative action of YKS (central nervous system or peripheral nervous system) toward cancer bone pain. Furthermore, it is also important to clarify which component of YKS plays a crucial role in alleviation of bone cancer pain and MMP-9 suppression. It has been recently reported that the components of* Atractylodis Lanceae* Rhizoma, one of the constituent herbal medicines in YKS, may play an important role in the regulation of IL-6 expression and neuropathic pain control [8]. However, which crude drug or pharmacologically active ingredient played the role in the suppression of MMP-9 and alleviation of cancer pain remains unknown. Further analysis is needed in the future.

In conclusion, in this mouse model of bone cancer pain YKS significantly lessened cancer pain behavior by suppressing MMP-9 expression, suppressing the expression in bone tissue in the early phase and in the spinal cord in the late phase of pain development.

## Data Availability

The experimental data used to support the findings of this study are included within the supplementary information files.

## Supplementary Materials

Supplementary Materials Sup.Table 1: time courses of cancer pain assessed by DWB. Sup.Table 2: dose dependencies of YKS. Sup.Table 3: effect of daily oral YKS. Sup.Table 4: the effect of i.t. or i.p. injection of saline, MMP-2 inhibitor, or MMP-9 inhibitor. Sup.Table 5: RT-PCR analysis. Sup.Table 6: immunostaining in the spinal cord.
