# Corrigendum to “Yokukansan Alleviates Cancer Pain by Suppressing Matrix Metalloproteinase-9 in a Mouse Bone Metastasis Model”

**DOI:** 10.1155/2019/3513064

**Published:** 2019-11-13

**Authors:** Kenta Nakao, Atsushi Fujiwara, Nobuyasu Komasawa, Denan Jin, Manabu Kitano, Sayuri Matsunami, Shinji Takai, Seiji Ito, Toshiaki Minami

**Affiliations:** ^1^Department of Anesthesiology, Osaka Medical College, 2-7 Daigaku-machi, Takatsuki, Osaka 569-8686, Japan; ^2^Department of Innovative Medicine, Graduate School of Medicine, Osaka Medical College, 2-7 Daigaku-machi, Takatsuki, Osaka 569-8686, Japan

In the article titled “Yokukansan Alleviates Cancer Pain by Suppressing Matrix Metalloproteinase-9 in a Mouse Bone Metastasis Model” [1], there was an error in Figure 2(c) in which the right-hand panel was duplicated in the left-hand panel during the preparation of the manuscript, which should be corrected as follows:

## Figures and Tables

**Figure 1:**
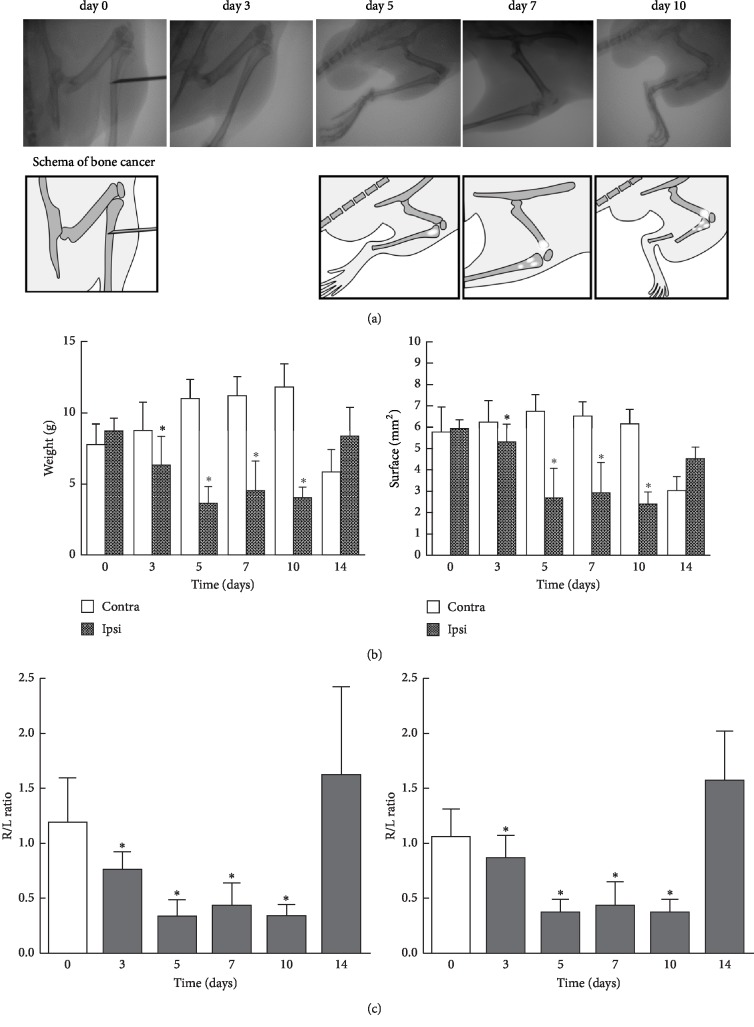
Preparation of bone cancer pain mice and time courses of cancer pain as assessed by weight bearing and surface resting of hind paws. (a) Radiographs of progressive bone destruction. These images are representative of the stages of bone destruction in the right tibia. (b, c) Animal body weight bearing and surface resting of right and left hind paws were measured for 5 min on the indicated days with the DWB device as described in “Materials and Methods.” Day 0 represents the presurgical baseline data. The data are expressed as the mean ± SD (n = 6 except for n =4 on day 14). One-way repeated ANOVA was applied for statistical analysis. *∗*P < 0.05 compared to left paw (b); *∗*P < 0.05 compared to the R/L ratio of day 0 (c) [label ordinate for the right-hand graph].
